# Multiscale Currents Observed by MMS in the Flow Braking Region

**DOI:** 10.1002/2017JA024686

**Published:** 2018-02-20

**Authors:** Rumi Nakamura, Ali Varsani, Kevin J. Genestreti, Olivier Le Contel, Takuma Nakamura, Wolfgang Baumjohann, Tsugunobu Nagai, Anton Artemyev, Joachim Birn, Victor A. Sergeev, Sergey Apatenkov, Robert E. Ergun, Stephen A. Fuselier, Daniel J. Gershman, Barbara J. Giles, Yuri V. Khotyaintsev, Per‐Arne Lindqvist, Werner Magnes, Barry Mauk, Anatoli Petrukovich, Christopher T. Russell, Julia Stawarz, Robert J. Strangeway, Brian Anderson, James L. Burch, Ken R. Bromund, Ian Cohen, David Fischer, Allison Jaynes, Laurence Kepko, Guan Le, Ferdinand Plaschke, Geoff Reeves, Howard J. Singer, James A. Slavin, Roy B. Torbert, Drew L. Turner

**Affiliations:** ^1^ Space Research Institute Austrian Academy of Sciences Graz Austria; ^2^ Laboratoire de Physique des Plasmas CNRS/Ecole Polytechnique/UPMC Univ Paris 06/University Paris‐Sud/Observatoire de Paris Paris France; ^3^ Earth and Planetary Sciences Tokyo Institute of Technology Tokyo Japan; ^4^ Department of Earth, Planetary and Space Sciences University of California Los Angeles CA USA; ^5^ Space Science Institute Boulder CO USA; ^6^ Earth's Physics Department St. Petersburg State University St. Petersburg Russia; ^7^ Laboratory for Atmospheric and Space Physics University of Colorado Boulder CO USA; ^8^ Southwest Research Institute San Antonio TX USA; ^9^ NASA, Goddard Space Flight Center Greenbelt MD USA; ^10^ Swedish Institute of Space Physics Uppsala Sweden; ^11^ Royal Institute of Technology Stockholm Sweden; ^12^ Applied Physics Laboratory Johns Hopkins University Laurel MD USA; ^13^ Space Research Institute (IKI) RAS Moscow Russia; ^14^ Department of Physics Imperial College London London UK; ^15^ LANL, CSES Los Alamos NM USA; ^16^ NOAA Space Weather Prediction Center Boulder CO USA; ^17^ Department of Climate and Space Sciences and Engineering University of Michigan Ann Arbor MI USA; ^18^ Institute for the Study of Earth, Oceans, and Space University of New Hampshire Durham NH USA; ^19^ Space Sciences Department Aerospace Corporation Los Angeles CA USA

**Keywords:** Magnetospheric Multiscale (MMS), field‐aligned current, flow braking, magnetic reconnection, plasma sheet boundary

## Abstract

We present characteristics of current layers in the off‐equatorial near‐Earth plasma sheet boundary observed with high time‐resolution measurements from the Magnetospheric Multiscale mission during an intense substorm associated with multiple dipolarizations. The four Magnetospheric Multiscale spacecraft, separated by distances of about 50 km, were located in the southern hemisphere in the dusk portion of a substorm current wedge. They observed fast flow disturbances (up to about 500 km/s), most intense in the dawn‐dusk direction. Field‐aligned currents were observed initially within the expanding plasma sheet, where the flow and field disturbances showed the distinct pattern expected in the braking region of localized flows. Subsequently, intense thin field‐aligned current layers were detected at the inner boundary of equatorward moving flux tubes together with Earthward streaming hot ions. Intense Hall current layers were found adjacent to the field‐aligned currents. In particular, we found a Hall current structure in the vicinity of the Earthward streaming ion jet that consisted of mixed ion components, that is, hot unmagnetized ions, cold E × B drifting ions, and magnetized electrons. Our observations show that both the near‐Earth plasma jet diversion and the thin Hall current layers formed around the reconnection jet boundary are the sites where diversion of the perpendicular currents take place that contribute to the observed field‐aligned current pattern as predicted by simulations of reconnection jets. Hence, multiscale structure of flow braking is preserved in the field‐aligned currents in the off‐equatorial plasma sheet and is also translated to ionosphere to become a part of the substorm field‐aligned current system.

## Key Points

- Multiscale field‐aligned currents in the boundary of the expanding plasma sheet during plasma jet braking intervals are resolved
- Intense Hall current layers are found at the inner boundary of the hot Earthward streaming ion jets and flow shear regions
- Both plasma jet diversion and Hall effects from reconnection region contribute to the structure of the substorm wedge currents

## 1 Introduction

The most dramatic energy release in the near‐Earth magnetotail is considered to be driven by the near‐Earth magnetic reconnection‐associated flows, called bursty bulk flows (BBFs), and electromagnetic disturbances propagating Earthward and interacting with plasmas in the near‐Earth dipolar region. In particular, during substorms, these disturbances lead to drastic changes in the magnetospheric configuration such as thinning and expansion of the plasma sheet, magnetic field dipolarization, and energetic particle injection. Enhanced coupling to the ionosphere results in strong currents, for example, auroral electrojets, field‐aligned currents (FACs), and auroral precipitations. The large‐scale current system during the substorm expansion phase, called the substorm current wedge (SCW) (McPherron et al., 1973), consists of a net FAC toward the magnetosphere out from the ionosphere (upward FAC) at the western edge and into the ionosphere at the eastern edge of the auroral activity, which is connected by a westward horizontal current in the ionosphere. A similar FAC pattern was also identified during localized magnetospheric disturbances such as pseudobreakups (Nakamura et al., 2001; Palin et al., 2015) as well as during BBFs (e.g., Henderson et al., 1998; Nakamura et al., 2001).

The occurrence frequency of Earthward BBFs or the rapid flux transport rate (enhanced dawn‐to‐dusk electric field) significantly drops inward of 10–15 R_E_ (e.g., Schödel et al., 2001), called the flow braking region, where significant energy dissipation and current disruption (unloading) processes take place (e.g., Sergeev et al., 2012, and references therein). It is the flow‐braking region where larger E_Y_ than in the midtail region was detected (Liu et al., 2014; Schmid et al., 2016; Tu et al., 2000), indicating that the flux transport rate itself may increase before the flows brake. A large dawn‐to‐dusk electric field is obtained also in magnetohydrodynamic (MHD) simulations due to the induced electric field in the flow‐braking region (Birn et al., 2011; Birn & Hesse, 1996). Vortex flows, oscillatory behavior of the flows (e.g., Panov et al., 2013), and small‐scale current sheet processes that break the frozen‐in condition have been reported in this region (e.g., Lui, 2013), suggesting that the flow braking involves dynamic multiscale processes.

On 10 August 2016 an intense (AL ~−1,000 nT) substorm was initiated at 09:57 universal time (UT) when multiple spacecraft were distributed at radial distances between 4 and 15 R_E_ in the nightside magnetosphere. The four spacecraft of the Magnetospheric Multiscale (MMS) were located in the southern hemisphere outer plasma sheet and observed fast flow disturbances associated with multiple dipolarizations. Nakamura et al. (2017) (hereafter referred to as Nak17) studied the large‐scale evolution of the current wedge based on the multiple spacecraft in the nightside magnetosphere and ground‐based observations. Several intense FAC layers were identified from MMS observations. Based on the analysis of the flows and motion of these FACs in comparison with other spacecraft and MHD simulations (Birn & Hesse, 2014), it was concluded that MMS encountered a FAC system at the high‐latitude side of the near‐Earth flow‐braking region. These observations show that the processes of Earthward flow braking as well as accumulated magnetic flux evolving tailward, which has been detected in the center of the plasma sheet (Nakamura et al., 2009), can be also detected at the boundary region of the near‐Earth plasma sheet. In this paper we study the characteristics of each FAC layer as well as the perpendicular currents observed by MMS. Using high‐resolution ion and electron data, particle population responsible for the currents is examined to understand the physical processes of these active plasma boundaries.

## 2 Overview of the Event

**Figure 1:**
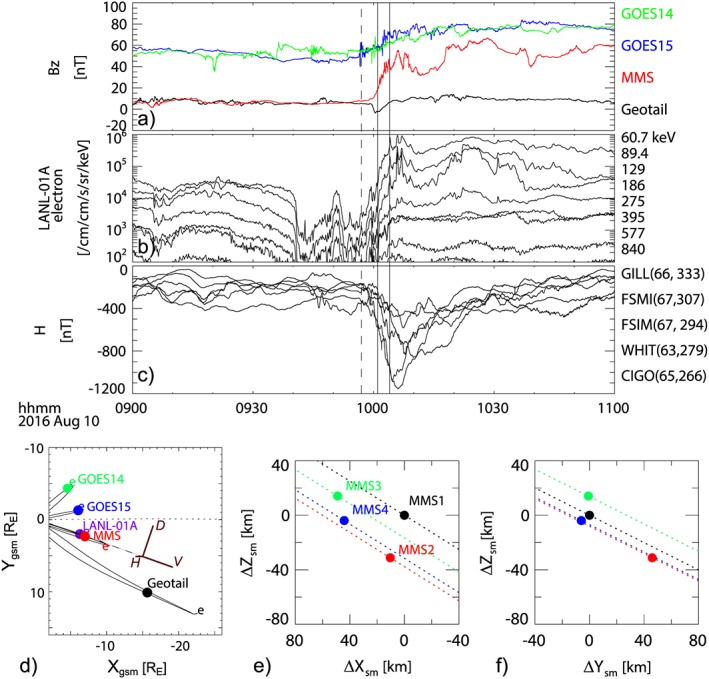
Overview of the 10 August 2016, 0957 universal time (UT) substorm. (a) Bz component of the magnetic field obtained from Geostationary Operational Environmental Satellite (GOES) 14 (green), GOES 15 (blue), Magnetospheric Multiscale (MMS) (red), and Geotail (black); (b) electron differential flux from LANL‐01A; (c) horizontal component of the geomagnetic field from ground stations; (d) location of the different spacecraft in the equatorial plane. Relative location of MMS spacecraft in (e) *x*‐*z* and (f) *y*‐*z* planes. The dashed line in (a)–(c) shows the 09:57 UT substorm onset, and the solid lines indicate 10:01 and 10:04 UT, which is the time interval of interest in this study. The curves drawn in (d) are the projected magnetic field lines from T89 model, and equatorial location is marked with “e.” Projected direction of the axis of the VDH coordinate system for MMS is given also in (d). The dotted lines in (e) and (f) show the satellite trajectories.

On 10 August 2016, MMS crossed the near‐Earth tail region when an intense substorm with multiple intensifications in the electrojet (Figure 1) commenced at 09:57 UT. The Bz component of the magnetic field (Figure 1a), obtained from Geostationary Operational Environmental Satellite (GOES) 14–15 (Singer et al., 1996), MMS (Russell et al., 2016), and Geotail (Kokubun et al., 1994), shows that multiple dipolarizations were observed by these spacecraft associated with an energetic particle injection (Figure 1b) and an intense electrojet enhancement (Figure 1c). Based on the analysis of the magnetic field disturbances at GOES 14 and 15, MMS, Geotail, and ground‐based magnetic field observations, it was shown that an SCW developed in the nightside region. MMS was located at the dusk part of the SCW (Nak17), close to the geosynchronous satellites, GOES 15 and LANL01A. The projected magnetic field lines from the T89 model (Tsyganenko, 1989) (Figure 1d) show that MMS was on a field line that crosses the equator about 3 R_E_ tailward of LANL‐01A. Since MMS was in the near‐Earth region, we will use the following coordinate systems in reference to the Earth's dipole, that is, the solar magnetic (SM) system or VDH coordinate system (explained below). The direction of the VDH axis for MMS, projected on the geocentric solar magnetospheric (GSM) equatorial plane, is given in Figure 1d. The location of the four MMS spacecraft in the SM system was (*x*_SM_, *y*_SM_, *z*_SM_) = (−6.7, 2.4, −2.2) R_E_ in the premidnight (22.7 magnetic local time) region. The spacecraft separation was about 50 km, as shown in Figures 1e and 1f. In this paper, we examine the characteristics of the FAC between 10:01 and 10:04 UT (the time interval indicated by the solid line) during the main part of the substorm expansion phase. During this time MMS was located at the western edge of a localized SCW, obtained based on analysis of low‐altitude ground based data and disturbances of magnetic field data from GOES and Geotail (NAK17).

**Figure 2:**
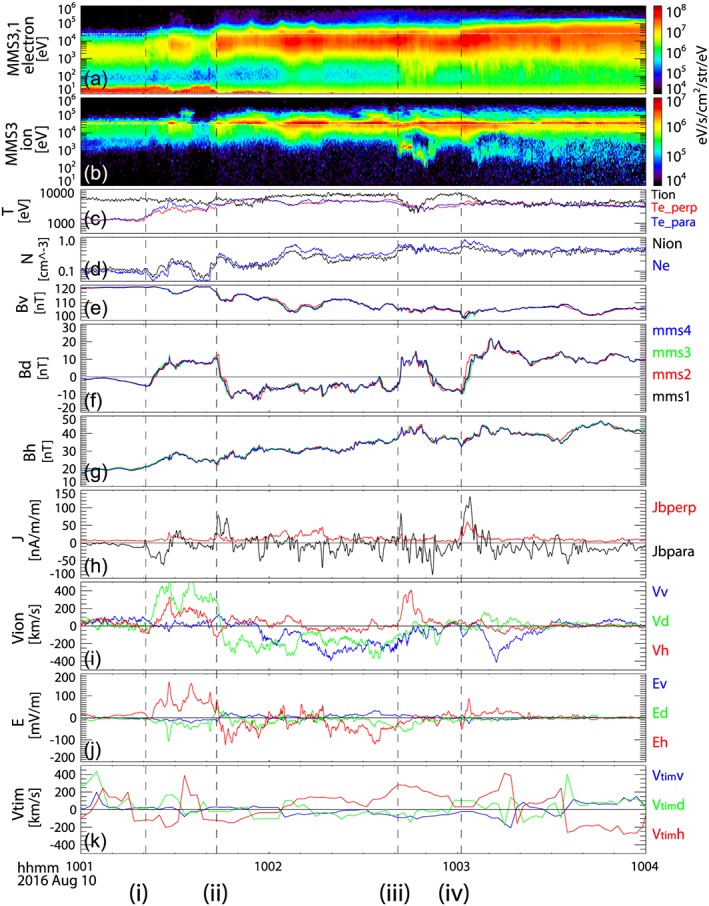
Magnetospheric Multiscale observations between 10:01 and 10:04 on 10 August 2016. Energy spectra from (a) electrons and (b) ions obtained from Energetic Ion Spectrometer (EIS) and Fast Plasma Instruments (FPI). Electron spectra for energy lower/higher than 25 keV are plotted using FPI and EIS data. The EIS electron energy flux is multiplied by 2.75. Ion spectra for energy higher/lower than 30 keV are plotted using EIS/FPI data. (c) Electron perpendicular (red) and parallel (blue) temperatures, (d) ion (black) and electron (blue) density, and (e) *V*, (f) *D*, and (g) *H* components of the magnetic fields from the four MMS spacecraft. (h) The parallel (black) and perpendicular (red) components of the currents determined using the curlometer method. *V* (blue), *D* (green), and *H* (red) components of the (i) ion flow, the (j) electric field, and the (k) timing velocity. Plasma moments are calculated using FPI data. The vertical dashed lines show the start of the crossing times of the main current layers associated with the dipolarization: (i) 10:01:22, (ii) 10:01:43, (iii) 10:02:41, and (iv) 10:03:01.

Figure 2 shows the plasma and magnetic observations from MMS between 10:01 UT and 10:04 UT. Electron and ion energy spectra from the MMS Energetic Ion Spectrometer (EIS) (Mauk et al., 2014) and the Fast Plasma Instruments (FPI) (Pollock et al., 2016) are shown in Figures 2a and 2b. The electron energy spectra are a combined product from the MMS 1 EIS instrument for energy >25 keV and from the MMS 3 FPI instrument for energy <25 keV (panel 2). To enhance the visibility of the high‐energy part, the EIS electron energy flux is multiplied by 2.75. The ion energy spectrum shown in Figure 2b is from MMS 3 and is a composite of the proton data from EIS for higher energies (>30 keV) and ion data from FPI for lower energies (<30 keV). For the FPI ion data, the background noise due to energetic electrons is subtracted. Here we first obtained the background counts, which are expected to be energy independent and are assumed to be the counts from the lowest energy channels (five lowest, typically), where we expect no actual plasma to be measured. The average counts are calculated for these low‐energy channels, and then these constant counts are removed from all the energy channels for each data sampling (150 ms). The distribution functions and moments are then calculated from the background‐subtracted data. The electron temperature and ion and electron densities from FPI are shown in Figures 2c and 2d. MMS was located in the outer plasma sheet until around 10:01:30 UT, then entered into a hotter and denser plasma sheet region, as can be seen in the plasma density and the electron temperature. The ion energy spectra show that significant ion population shift to the energy of EIS so that the latter contribution becomes important in particular for temperature (not shown).

The entry into the hotter plasma sheet is associated with multiple dipolarizations, as can be seen in the magnetic field data from the four MMS spacecraft shown in Figures 2e–2g. Here we used the VDH coordinate system, since it better represents the magnetic disturbance in a dipolar configuration at a local time away from the midnight region. *H* is the same as the *z* component in the SM along the geomagnetic dipole axis and is positive northward. *D* is perpendicular to *H* and the radial direction, *R*, and is positive eastward. *V* closes the right‐hand coordinate system and is positive in the radially outward direction. For this event, therefore, +*V* and +*D* directions at MMS location correspond to approximately −*x*_SM_ and −*y*_SM_ directions with about 19° clockwise rotations viewed from the north. The coordinate system is convenient for this event, since the background field parallel to the current sheet is nearly aligned to the *V* direction. The overall decrease in *B*_*V*_ (Figure 2e) and increase in *B*_*H*_ (Figure 2g) indicate the change from a tail‐like to a more dipolar configuration associated with the expansion of the plasma sheet. The main disturbances during these events are the step‐like changes in the *B*_*D*_ component (Figure 2f) due to FACs, which is also confirmed by the current profile (Figure 2h). Jb_perp and Jb_para shown in Figure 2h are currents perpendicular and parallel to the magnetic field and are deduced by applying the curlometer method (Chanteur, 1998) to the magnetic field data from the four MMS spacecraft. The four main sharp changes in *B*_*D*_, corresponding to the intense FAC layers, are all accompanied by dipolarization fronts (*B*_*H*_ enhancements). The dashed lines mark the start of these events: (i) 10:01:22, (ii) 10:01:43 (iii) 10:02:41, and (iv) 10:03:01. The FAC during event (i) was antiparallel to the magnetic field direction; that is, currents are flowing into the ionosphere (downward FAC). The direction of the FACs of events (ii)–(iv) was parallel to the magnetic field; that is, currents are flowing out from the ionosphere (upward FAC).

Figure 2i depicts the 1 s averaged FPI ion flow data, showing that the FAC events are related to enhancements in the high‐speed flows, mainly in the dawn‐dusk direction. Event (i) corresponds to enhancements in dawnward (+Vd)/northward (+Vh) flow. The flow changed to duskward (−Vd) during event (ii), followed by an enhancement in Earthward flow (−Vv). Event (iii) is associated with enhancement in northward (equatorward) flow direction, while event (iv) is followed by enhancement in Earthward (−Vv) flow. Note that the velocity moment, which is calculated using FPI with its limited energy range (<30 keV), does not necessarily reflect the ion bulk flow, in particular after the entry to hot plasma sheet. We therefore examine the ion distribution function and compare the ion moments with the *E* × *B* drift as well as with the current calculations from the magnetic field data in order to characterize the ion behavior in more detail later. Nonetheless, the overall direction of the ion bulk flow described above and the existence of the high‐speed flows during this interval are still valid.

The 1 s averaged electric field obtained from the electric field double probe instrument (Ergun et al., 2016; Lindqvist et al., 2016) from MMS 3 is shown in Figure 2j. Intense electric fields, mainly in north‐south direction with some enhancements in the duskward component, were observed during the FAC intervals. The enhancement of the northward electric field is associated with FAC event (i), and the southward turning of the electric field is associated with FAC event (ii). Event (iii) took place not only during the recovery of the southward electric field but also during the largest enhancement in duskward electric field (−Ed). Event (iv) was observed associated with short time scale enhancement in the electric field, first southward then northward.

Since the changes in the *B*_*D*_ component of the magnetic fields dominate among the other components in Figures 2e–2g associated with the FAC events (i)–(iv), a planar FAC sheet is a valid assumption, which expected to be in the near‐Earth PSBL, and the FAC is flowing approximately along *V* direction. In order to estimate the overall motion of the PSBL we applied the timing methods (see Schwartz, 1998, and reference therein). With the timing method orientation and motion of a planar boundary can be obtained using observation from the four spacecraft with some time differences. That is, observations from spacecraft pairs *α* and *β* with a separation vector, **r** *αβ*, are used to determine the time difference *tαβ*, where *α* = 1 and *β* = 2, 3, and 4. The speed of the magnetic structure, which we call “timing velocity,” **V**_tim_ = V_tim_ · **n**, can then be determined by obtaining the solution of

(1) $$\left(\begin{array}{c} r_{12}\\ r_{13}\\ r_{14} \end{array}\right)\cdot \left(\begin{array}{c} n_{x}\\ n_{y}\\ n_{z} \end{array}\right)=V_{\mathrm{tim}}\left(\begin{array}{c} t_{12}\\ t_{13}\\ t_{14} \end{array}\right)$$

In order to examine the overall motion of the PSBL, we estimated the timing velocity every 2 s, and the results are shown in Figure 2k. Here time differences between pairs of spacecraft are determined by cross‐correlation of the *B*_*D*_ over time interval of 10 s. Since VDH is a local coordinate system, which differs for each spacecraft, we determine first the timing velocity in SM coordinate system and then transformed into the VDH coordinate system at MMS 3. The normal component of PSBL or FAC layer is expected to be approximately the *H* component so that the motion of the FAC/PSBL will appear in the *H* component of *V*_tim_. Although we have fixed the time scales of the magnetic structure to 10 s with this method, the overall motion of the PSBL is well reconstructed. That is, outward motion of the plasma sheet (negative *V*th) during events (i) and (ii) followed by inward motion during events (iii) and (iv), and then later again outward motion of the plasma sheet when the Earthward flow subsides.

**Figure 3:**
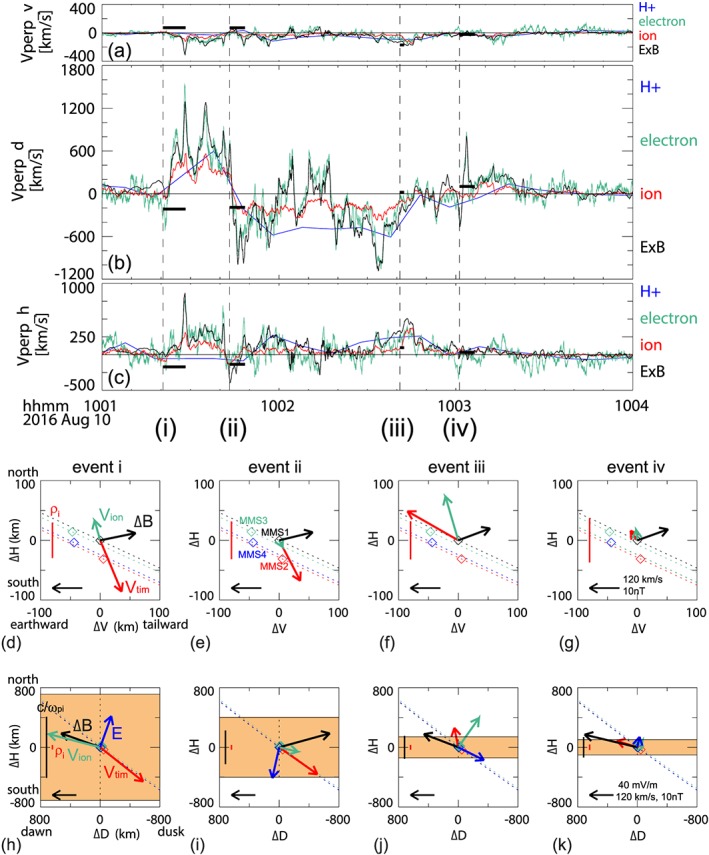
Motion of the plasma and the boundary structures during the four field‐aligned current (FAC) events. (a) *V*, (b) *D*, and (c) *H* components of the flows perpendicular to the magnetic field. The black curves correspond to *E* × *B* drift, the red and green curves are the Fast Plasma Instruments ion and electron velocities, and the blue curves are the proton velocity from Hot Plasma Composition Analyzer. The horizontal black bars in (a)–(c) represent the timing velocities, and the vertical dashed lines indicate the start times of events (i)–(iv). The average direction of the field and flow disturbances during the events (i)–(iv) are summarized in (d)–(k). The average ion flows perpendicular to magnetic field (Vion, green), the timing velocity vector (Vtim, red), and the magnetic field disturbance vector (Δ*B*, black) during the four current sheet crossings (i–iv) are shown in (d)–(g) projected on the *V*‐*H* plane and in (h)–(k) projected on the *D*‐*H* plane. The electric field is also shown in dark blue in (h)–(k). The spatial scales: on ion‐gyro radius (red) and one inertial length (black), are shown as vertical bars. The estimated thickness of the FAC sheet is presented as orange area. The relative locations of the spacecraft to MMS 1 are also given together with the orbit direction (dashed line) in (d)–(g).

In Figures 3a–3c the *V*, *D*, and *H* components of *E* × *B* drift velocity (black curves) are compared with the plasma flow velocity perpendicular to the magnetic field obtained from FPI and Hot Plasma Composition Analyzer (HPCA; Young et al., 2014) measurements. While the HPCA scans plasmas up to 40 keV for different ion species, the energy channel of FPI ions and electrons are up to 30 keV. The *E* × *B* drift velocity and the electron (green curves) and ion (red curves) velocity data are boxcar‐averaged over 0.6 s. The time resolution of the proton velocity (blue curve) from HPCA is 10 s. As expected from the northward and then southward electric field, the overall flow direction was changing from the dawnward to duskward direction during event (i) and event (ii) and then recovered after event (iii). The ion velocity from FPI with lower energy coverage is mostly lower than the HPCA velocity and is likely underestimated, in particular after entering the plasma sheet proper, as discussed before. Nonetheless, it can be seen that the overall changes in the flow direction are similar among the four velocity estimates. Taking into the different time scale of the changes in *B*_*D*_, the timing velocity, *V*_tim_, was estimated for each event (Nak17) and shown as the horizontal bar in Figures 3a–3c. They are obtained using the change in the *B*_*D*_ among the four spacecraft during the interval plotted by the horizontal bar in Figures 3a–3c. For events (i) and (ii), the direction of *V*_tim_ is southward (outward), tailward, and duskward and opposite for events (iii)–(iv). Plasma is moving northward/equatorward with respect to the boundary as expected in the enhanced dawn‐to‐dusk convection electric field (−Ed) for events (i)–(iii), while the FAC event (iv) took place after the convection electric field enhancement (see Figure 2j). The timing method assumes a planar boundary with a stable structure moving with one constant speed, and hence, it is an average speed over the entire current sheet neglecting some internal structures. The boundary motion can reflect however also a temporal evolution of the current sheet such as the case for expansion of the plasma sheet. The electric field/plasma should reflect the motion of the overall current sheet and the equatorward convection as well as the internal processes within the current sheet. Therefore, an apparent difference between *V*_tim_ and plasma motion can take place during events (i) and (ii) when plasma sheet expands outward while plasma is convecting equatorward.

The average ion velocity vector, *V*_ion_ (green), *V*_tim_ (red), and the magnetic field difference, Δ*B* (black), between the start time and end time of the FAC crossing interval, are shown projected on the *V*‐*H* plane in Figures 3d–3g and on the *D*‐*H* plane in Figures 3h–3k. The electric field, *E* (blue), is also shown in Figures 3h–3k. Note that these FACs (flowing mainly along *V*) are producing the magnetic disturbances predominantly along *D*, as can be seen in the black arrow, Δ*B*, and hence, the timing velocity along the *H* direction represents the motion of the current sheet. The spatial scale of the four current sheets was estimated using the *H* component of the *V*_tim_ and the duration of the *B*_*D*_ change (Nak17), which is highlighted as orange region in the bottom panels. The thicknesses of these currents sheets are (i) 1310 km, (ii) 710 km, (iii) 140 km, and (iv) 170 km. The black vertical bars correspond to the ion‐inertial scale, while the red vertical bar shows the ion‐gyro radius. It can be seen that the spatial scale of the FAC events (i) and (ii) are well above the ion scales, whereas events (iii) and (iv) are comparable to or less than the ion‐inertial scale and a couple of ion‐gyro scales. Note that the relative thickness to the ion gyro radius may be smaller for events (iii) and (iv) due to possible underestimation of ion thermal velocity during these times. Hence, at least for events (i) and (ii), the overall pattern of the intense dawnward flow and reversal to duskward flows and associated FACs are expected to be a large‐scale process, although some ion‐scale substructures are embedded in these events (see Le Contel et al., 2017 for detail). Based on these flow and field patterns at MMS, observations from ground and other spacecraft, and comparisons with an MHD simulation, NAK17 suggested that events (i) and (ii) detected at MMS are most likely due to the crossing of the high‐latitude side of the current wedge produced in the flow‐braking region.

In addition to the overall dawn‐dusk reversals that are seen in the ion, electron, and *E* × *B*‐drift velocities, there are shorter time‐scale disturbances detected in the *E* × *B* (and electron) flows when there is a significant deviation between them and the ion (and proton) velocity. In these plots, all velocities are averaged over 1 s, which is still larger than the average ion gyroperiod of ~0.6 s. The electron perpendicular velocity changes follow most of the time with the *E* × *B* drift changes. There are also sharp peaks when the electron and *E* × *B* drifts deviate, suggesting the existence of smaller scale processes. Around event (iv), the dawn‐dusk flow reversals show very good coincidence between the electron and the *E* × *B* drift under the condition of nearly zero ion velocity, indicating that the profile is due to Hall effects as will be discussed more detail in section 4.

## 3 FAC Layers and Characteristics of Electrons

**Figure 4:**
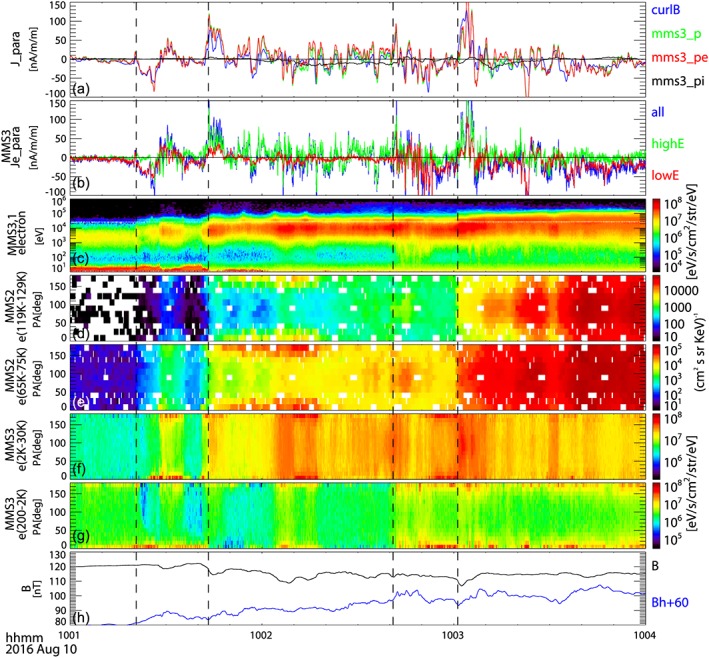
Parallel currents and spectral characteristics of electrons. (a) Parallel current density estimated using fluxgate magnetometer data from the four Magnetospheric Multiscale (MMS) applying the curlometer method (blue), currents calculated from ions (black) and electrons (red), and total of ions and electrons (green) from Fast Plasma Instruments (FPI) measurements of MMS 3. (b) Electron parallel currents from FPI for different energy range: higher energy (2–30 keV) shown in green, lower energy (200 eV–2 keV) shown in red, and all energies shown in blue. (c) Electron energy spectra using same format as in Figure 2a. Electron pitch angle spectra for the energy range of (d) 119–129 keV and (e) 65–75 keV obtained by the MMS 2 energetic particle detector/fly's eye energetic particle spectrometer instrument and for (f) 2–30 keV and (g) 200 eV–2 keV obtained by MMS 3 FPI measurement. (h) Magnitude (black) and *B*_*H*_ (blue) component (added by 60 nT) of magnetic field from MMS. The vertical lines indicate the four field‐aligned current events.

In order to examine the characteristics of the FAC layers, we examine the plasma population responsible for carrying the current and compare with the curlometer currents. Figure 4 shows the parallel currents and electron pitch angle distribution (PAD). The parallel current (Figure 4a), J_para, obtained by curlometer (labeled as curlB, blue curve) agrees well with the total plasma (mms3_p, green) current, which is predominantly carried by electrons (mms3_pe, red). Hence, for the parallel current the effect of possible underestimation of the ion current (mm3_pi, black) due to the limited energy range is negligible as the parallel electron velocity is much larger than the parallel ion velocity. The electric current data shown in Figure 4a are averaged over 0.5 s to be comparable to the ion‐gyro period. The particle currents are calculated from MMS 3 data. The curlometer current, on the other hand, approximates the current at the barycenter and is obtained assuming linear gradients in *B* over the spatial scale of MMS, that is, about 50 km. The overall agreement between the particle and curlometer current density profiles for the four FAC current sheets therefore suggests that the four current sheets have spatial scale sizes of ions or larger, as expected from the estimation of the current sheet scales shown in Figures 4h–4k. Yet electron currents show additional short‐time scale fluctuations such as those seen in the later part of event (iv). In fact, the electron currents parallel to the magnetic field, Je_para, are highly structured during most of the interval except for event (i) as shown in Figure 4b, wherein the electron parallel currents are depicted using 0.1 s averaged data from FPI for different energy range: higher energy (2–30 keV) shown in green, lower energy (200 eV–2 keV). Figures 4c–4g show changes of the energy and PAD of electrons for the selected energy ranges. Pitch angle spectra are shown for energy range of 119 keV–129 keV (Figure 4d) and 65 keV–75 keV (Figure 4e), obtained from the energetic particle detector/fly's eye energetic particle spectrometer instrument (Blake et al., 2016) aboard MMS 2 depicting the nonthermal electron pitch angle signatures and for 2 keV–30 keV (Figure 4f) and 200 eV–2 keV (Figure 4g) obtained from FPI measurement aboard MMS 3 depicting the thermal part of the electron signatures.

The electrons responsible for upward and downward currents are quite different in their energy ranges, as can be seen in Figure 4b. In general, the higher‐energy population (2 keV–30 keV) contributes mainly to the upward (parallel to field line) current, while the lower energy (200–2 keV) electrons are the dominant carrier of the downward current during most of the times, though the populations associated with the short‐time scale fluctuations in the FAC are more variable. Since the Earthward fast flows indicate that MMS was Earthward of the near‐Earth reconnection region, the higher‐energy electron populations streaming Earthward (contributing to upward FAC) may correspond to electrons accelerated in the reconnection region (e.g., Hoshino et al., 2001) and due to Fermi‐type acceleration for nonthermal trapped population associated with the reconnection jet (e.g., Fu et al., 2011). Possible sources of the cold electrons streaming tailward (downward FAC) are ionospheric population accelerated by the kinetic Alfvén waves observed at PSBL (e.g., Wygant et al., 2000) and the lobe population accelerated along the field line due to the large‐scale electric field (Egedal et al., 2012) or localized double layer (Fujimoto, 2014) at the Earthward side of the reconnection region.

The four FAC events (i–iv) are associated with distinct changes in the energies of electrons as well as the PAD, resulting in stepwise enhancements in energy. FAC event (i) is associated with enhancements in the low‐energy parallel electrons (Figure 4g), producing the downward current. Event (i) is followed by upward FACs associated with enhancements in the higher‐energy electron (above few keV) population in the parallel/antiparallel direction (Figure 4f). It is interesting to note that around 01:28 UT there is a distinct downward current peak associated with parallel (tailward) moving energetic electrons up to 75 keV, as can be seen in Figure 4e. This electron signature requires therefore a tailward acceleration, opposite to the general trend mentioned above. More detail on this event is provided by Le Contel et al. (2017). FAC event (ii) is associated with enhancements in the antiparallel electrons of a few tens of keV (Figure 4f), followed by enhancements in the parallel and antiparallel portions of the electron population in the 65–75 keV range (Figure 4e). The latter two events, however, are associated with enhancements in suprathermal electrons centered at a 90° pitch angle, as can be seen in Figure 4d for event (iii) and Figure 4e for event (iv). The magnetic field magnitude during event (iv) increases with the dipolarization, indicating that this is likely a betatron effect.

## 4 Characteristics of Perpendicular Currents

**Figure 5:**
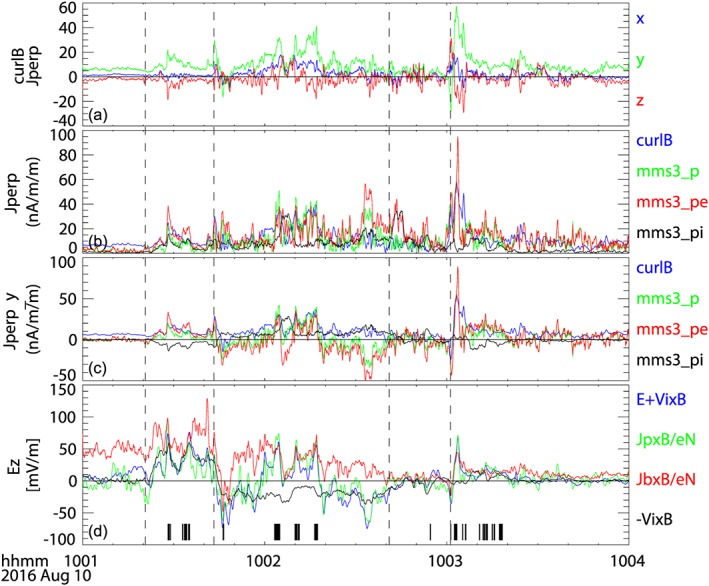
Perpendicular current density estimated using fluxgate magnetometer data from the four Magnetospheric Multiscale (MMS; curlometer method) and using Fast Plasma Instruments measurement from MMS 3. (a) *X*_GSM_ (blue), *Y*_GSM_ (green), and *Z*_GSM_ (red) components of the perpendicular current density obtained from the curlometer method. (b) Magnitude of the perpendicular current density. (c) *Y*_GSM_ component of the perpendicular current density estimated from the curlometer (curlB, blue), particle currents from ion and electron (mms3_p, green), from electron (mms3_pe, red), and from ion (mms3_pi, black). (d) *Z*_GSM_ components of the perpendicular electric field: *E* + Vi × *B* (blue), where *E* is the measured electric field double probe instrument electric field, Jp × *B*/eN (green), Jb × *B*/eN (red), and −Vi × *B* electric field (black). The black bars in (d) indicate times when the *E* + Vi × *B* coincides with both Jb × *B*/eN and Jp × *B*/eN within 30%. The vertical lines indicate the four field‐aligned current events.

Figures 5a–5c show the perpendicular current density estimated from the curlometer method and the particle measurements in the GSM coordinate system. As expected in the nominal magnetotail current sheet, the perpendicular component of the curlometer current, Jb_perp (panel a), shows that the current is predominantly in the dawn‐to‐dusk direction (green component), except for during short peaks such as the beginning of event (iv). The magnitude and *y*_GSM_ component of the perpendicular currents, J_perp and Jperp_y, deduced from particles and from the curlometer method are compared in Figures 4b and 4c, respectively. Total particle current (mms3_p, green) and curlometer current (curlB, blue) show mostly comparable values (Figure 5b). Yet the dawn‐dusk current shows some inconsistency in the direction during the interval when dawnward Jperp_y current was observed for particle current, while the curlometer current shows predominatly duskward current between events (ii) and (iii). These differences may likely come from the underestimation of the ion current (mms3_pi, black trace), which can be seen from the difference in the ion velocity obtained from FPI and that from HPCA during this interval as shown in Figure 3b. It can be seen that most of the transient enhancements in the dawn‐dusk component of the curlometer currents are associated with enhancements in the electron currents suggesting that these are the Hall current layers.

To examine the signatures of the Hall current in a more quantitative way, we examined the contribution of the Hall term in the generalized Ohm's law and shown in Figure 5d. Here we examined the *z* component, which is the main component of the electric field during this observation. Here we compare *E* + Vi × *B* (blue curve) with the Hall term calculated from the particle current, Jp × *B*/eN (green), and from the curlometer current, Jb × *B*/eN (red). Those times when *E* + Vi × *B* agrees both with Jb × *B*/eN and Jp × *B*/eN within 30% are marked with the tick marks at the bottom of Figure 5d. Many of the transient dawn‐dusk current enhancements shown in Figures 5a–5c are associated with the times when the Hall term dominates in the generalized Ohm's law, that is, *E* + Vi × *B* = *J* × *B*/eN = (Vi − Ve) × *B* ≠ 0. In order to compare the Hall electric field with the convection electric field, we plotted also (−Vi × *B*) as black trace in Figure 5d. The maximum Hall electric field, *J* × *B*/eN, from the curlometer current was 133 mV/m, which was about the double of the maximum convection electric field (−Vi × *B*), 66 mV/m, for the data shown in Figure 5d. The importance of the Hall effect, however, differs among the events. For example, the Hall electric field is about double the amplitude of the convection electric field (−Vi × *B*) for those times indicated by the tick marks between event (i) and (ii), but the Hall electric field completely dominates during the later events, in particular those around 10:03 UT.

**Figure 6:**
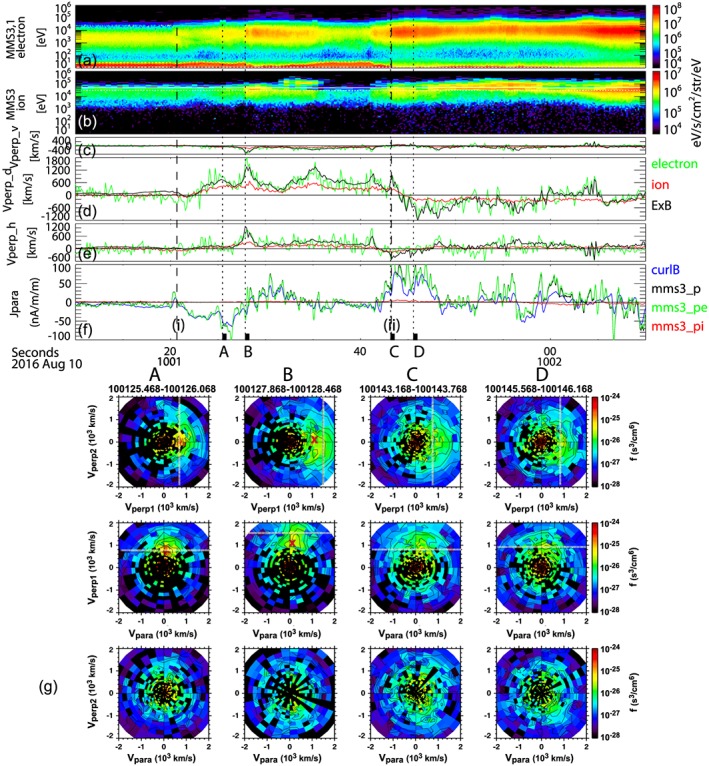
(a) Ion and (b) electron energy spectra; (c) *V*, (d) *D*, and (e) *H* components of the perpendicular velocities of particle and *E* × *B* drift; and (f) parallel current densities estimated by curlometer and plasma. (g) Ion distribution during the selected times: *t*_*A*_, *t*_*B*_, *t*_*C*_, and *t*_*D*_ marked at the bottom of (f), presented for three cuts in the Vperp1‐Vperp2 plane, in the Vpara‐Vperp1, and in the Vpara‐Vperp2 from top to bottom. *E* × *B* velocity is marked as a dashed line. “X” in the VDF cut *t*_*B*_ in (g) shows the location of the peak around Vperp1 discussed in the text.

The Hall current features are further examined using 2‐D cuts of the velocity distribution during selected times around FAC events (i) and event (ii) in Figure 6. The energy spectra, the *V*, *D*, and *H* components of the perpendicular velocity and parallel currents, Jpara, are also shown in Figure 6. We present the ion distribution functions averaged over four data points, that is, 0.6 s (approx. 1 ion gyroperiod), to enhance the count rate of the ions but still frequent enough to the resolve the ion‐scale feature. The velocity distribution functions (VDFs) are shown in the local field‐aligned coordinate system. Here we defined the Vperp1 by the *E* × *B* direction, Vperp2 by the *B* × (*E* × *B*) direction, and Vpara by *B*. The cut of the distribution function is the average of phase‐space densities within ±20° relative to the plotted plane. For the *E* and *B* directions, 0.6 s averaged electric field and magnetic field data are used. Note that the *E* × *B* direction coincides most of the time with the perpendicular velocity of the electrons (see Figure 3). Hence, the obtained VDF profiles in ion enable to examine the existence of Hall currents, to confirm the magnetized/unmagnetized signatures of the ions, to check whether the ions consist of single component or multicomponents, and to check the effect of the background.

During the FAC event (i) the *E* × *B* drift and ion and electron perpendicular velocities were enhanced as shown in Figures 6c–6e. As expected, the ion VDF at *t*_*A*_ (Figure 6g) has a peak in the direction of Vperp1 close to the *E* × *B* drift speed (vertical dashed line in the Vperp1‐Vperp2 panel and horizontal dashed line in the Vpara‐Vperp1 panel). The deviation between the *E* × *B* drift and ion velocity becomes larger around *t*_*B*_ (Figures 6c–6e), which can also be seen in the 2‐D cuts of VDF at *t*_*B*_ (Figure 6g). That is, there is a peak in VDF at *t*_*B*_, which is smaller than the *E* × *B* drift as can be seen in the location marked with a red “X” in the Vperp1‐Vperp2 and Vpar‐Vperp1 panels from *t*_*B*_, showing the peak along Vperp1 axis. The VDF peak at lower values than the *E* × *B* drift can be also seen in the VDF cut at *t*_*C*_ in the Vpar‐Vperp1 plane. The time *t*_*C*_ corresponds to FAC event (ii), just before the reversal of the dawn‐dusk flows. These deviations of the ion perpendicular velocity from the *E* × *B* drift, which was comparable to the electron perpendicular velocity, are expected for the Hall current layer. The peak in VDF at *t*_*D*_, on the other hand, coincides with *E* × *B* drift peak. That is, during this time, the difference between the ion and electron drifts, and thus, the Hall current should have been decreased, which is consistent with the low level of the curlometer current shown in Figure 5. These observations clearly show that there are ion‐scale Hall current layers embedded in the reversal region of the FAC layers between event (i) and event (ii) where ions move slower than *E* × *B* drift, that is, the electron motion.

**Figure 7:**
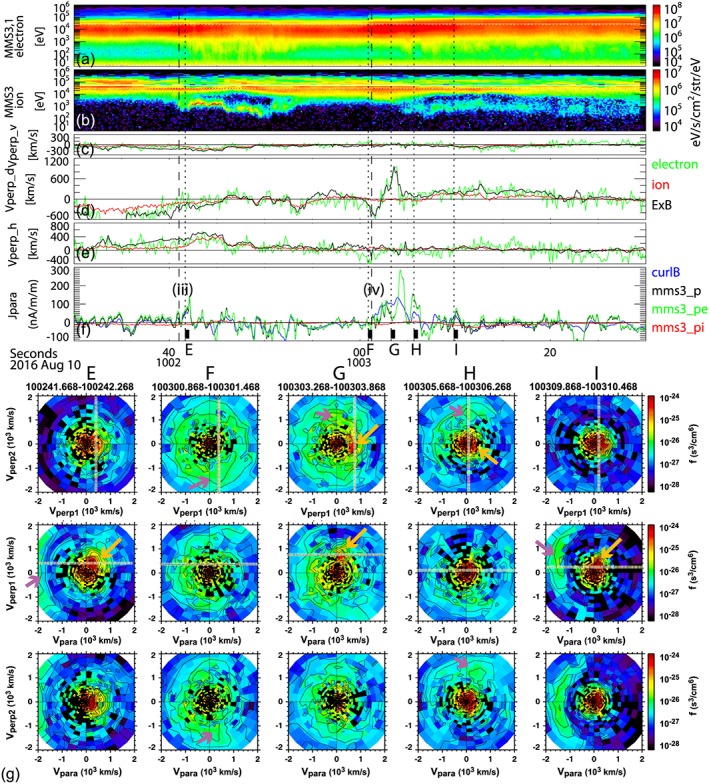
(a) Ion and (b) electron energy spectra; (c) *V*, (d) *D*, and (e) *H* components of the perpendicular velocities of particle and *E* × *B* drift; and (f) parallel current densities estimated by curlometer and plasma. (g) Ion distribution during the selected times: *t*_*A*_, *t*_*B*_, *t*_*C*_, and *t*_*D*_ marked at the bottom of (f), presented for three cuts in the Vperp1‐Vperp2 plane, in the Vpara‐Vperp1, and in the Vpara‐Vperp2 from top to bottom for ion spectra (g) and Vpara‐Verp1 for electron spectra. *E* × *B* velocity is marked as dashed line. Cold ion and hot ion components discussed in the text are indicated by yellow and pink arrows in (g).

Particle velocity distributions around the FAC events (iii) and (iv) are presented together with the energy spectra, perpendicular velocity, and parallel current in Figure 7 in the same format as Figure 6. The most significant difference in the ions for the FAC events (iii) and (iv) compared to the previous intervals shown in Figure 6 is the existence of the cold component. They become prominent, as can be seen in VDF plots, at *t*_*E*_ and *t*_*I*_ (Figure 6g, indicated by yellow arrows). For both events (iii) and (iv), hot ions tend to move antiparallel (Earthward, indicated by pink arrows in Figure 6g), while the cold ions are slowly moving parallel (tailward) suggesting that the hot ions are coming from the tail reconnection region and the cold ions from the direction of the ionosphere. The perpendicular velocity of the cold ions coincides well with the *E* × *B* drift as can be seen in Vpara‐Vperp1 plots in Figure 6g. As discussed before (Figure 3) the FAC current sheet of event (iv) is comparable to the ion‐inertia as well as a couple of thermal ion gyro radii, and corresponding nonmagnetized ion signatures can be seen in VDF from *t*_*E*_, *t*_*F*_, and *t*_*G*_ in the hot ion components. The VDF from *t*_*E*_ shows that the hot ion component is relatively isotropic, but instead of moving duskward (−V_D_) with the *E* × *B* drift, the distribution shows motion in the −Vperp2, −Vpara direction, corresponding to equatorward and Earthward motion. The subsequent large *E* × *B* drift enhancement in the dawnward (+V_D_) direction (Figure 6d) coincides well with the motion of the cold‐ion population seen in the Vperp1 direction in the VDF plots at *t*_*F*_. Again, the hot population does not drift with *E* × *B* but is distributed along the Vperp2 direction as expected in a thin current sheet of nonmagnetized ions, which can be seen most clearly in the VDF plots at *t*_*G*_. Hence, a slower perpendicular speed of the net ion population relative to that of the magnetized electron is expected. The net perpendicular current is therefore mainly the sum of the *E* × *B*‐drifting cold ions and electrons.

## 5 Discussion

Magnetospheric Multiscale encountered multiple intense FAC layers in the outer plasma sheet of the near‐Earth flow braking region during intense substorms (AE > 1,000 nT) on 10 August 2016. From multipoint data analysis of MMS, GOES 14 and 15, and Geotail in the nightside magnetosphere and ground‐based observations, Nak 17 studied the evolution of the SCW. Associated with expansion of the plasma sheet, MMS first encountered downward and upward field aligned currents (events i and ii). It was shown that the overall changes of the disturbances of flows and magnetic fields, and north‐south electric field, observed during events (i) and (ii) are similar to those predicted from the MHD simulation by Birn and Hesse (2014) of the near‐Earth flow braking of the localized reconnection flows (Nak 17). It is also suggested that the successively reconnected field lines can result in outward motion of the boundary (plasma sheet expansion) in the flux pileup region (FPR) in spite of enhanced inward plasma motion in the off‐equatorial outer plasma sheet such as shown in MHD simulation (Birn et al., 2013).

**Figure 8:**
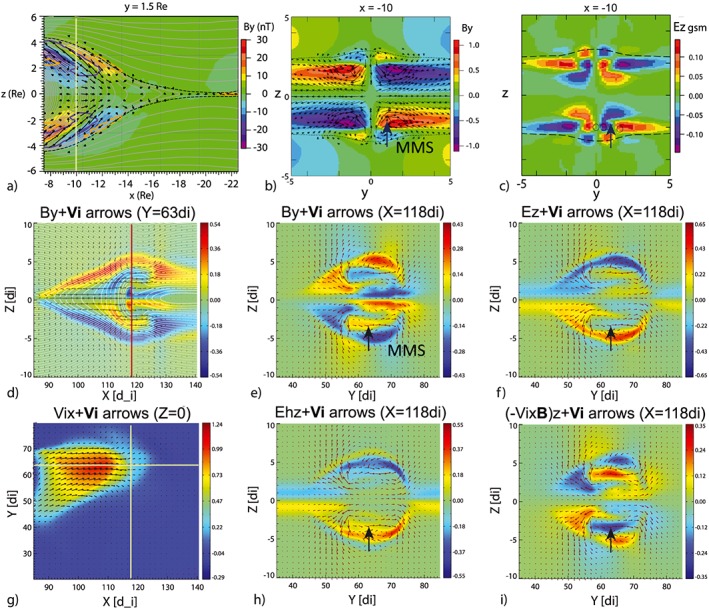
Reconnection jets in magnetotail magnetohydrodynamic (MHD) simulation by Birn and Hesse (2014) and in Hall‐MHD simulation by Nakamura et al. (2012). (a) Color‐coded By in *x*‐*z* plane at *t* = 132 at *y* = 1.5 together with the current density vectors (Δ*j*) akin to Figure 8a of Birn and Hesse (2014). The dark blue and orange contours indicate regions of enhanced tailward and Earthward field‐aligned currents, respectively. Color‐coded (b) By (normalized to 20 nT) and (c) vertical (northward) electric field Ez (normalized to 20 mV/m) in the *y*‐*z* plane at *t* = 132 at *x* = −10 from the same MHD simulation. (d) Color‐coded By in *x*‐*z* plane at *y* = 63 di, (e) By and (f) Ez in the *y*‐*z* plane at *x* = 118 di, (g) Vi in the equatorial plane, and (h) Ehz and (i) (−Vi × B)z in the *y*‐*z* plane at *x* = 118 di from Hall‐MHD simulation by Nakamura et al. (2012). Possible Magnetospheric Multiscale location and direction of the crossing during events (i) and (ii) in the *y*‐*z* plane are shown as black thick arrows in (b), (c), (e), (f), (h), and (i). The thin arrows in (c) and (d) show the *E* × *B* drift vectors, while the other vectors are Vi flows. The color scale of By shown in (d) and (e) is normalized to the initial magnetic field outside the current sheet, *B*_0_, and the Vix in (g) is normalized to the Alfvén velocity outside the current sheet, *V*_*A*_ ~*B*_0_/(*N*_0_)^½^, where *N*_0_ is the density outside the current sheet and Ez in (f), (h), and (i) is normalized to *V*_*A*_ *B*_0_.

While the MHD model proved the context of the overall observations (NAK17), transient disturbances and intense smaller‐scale current sheet signatures are prominent in the MMS observations. For example, several thin duskward/dawnward Hall currents were identified as shown in Figure 5. In order to discuss possible signatures to be observed around the outflows of reconnection (reconnection jets), when the Hall effect is considered, we refer to the simulation results in Figure 8 showing a localized jet in an MHD simulation by Birn and Hesse (2014) and in a Hall‐MHD simulation by Nakamura et al. (2012). The top three panels of Figure 8 show (a) By disturbances in the *x*‐*z* plane at the dusk part of a reconnection jet and (b) By and (c) Ez disturbances in the *y*‐*z* plane from the large‐scale MHD simulation by Birn and Hesse (2014), where the detailed setting is described. In this simulation, the near‐Earth reconnection region is formed at *x* ~−20 and a localized flow burst near midnight reaches the near‐Earth region *x* = −10 by *t* = 130. Figures 8b and 8c are taken from this time and location to show the effect of the dipolarization front and flow vortices surrounding the flow burst in the near‐Earth region, where MMS is expected to have been traversing (indicated by an arrow in Figure 8b). As discussed in Nak17, these patterns well represent the overall observation associated with the expansion of the plasma sheet. That is, the direction of the dawn and then duskward perturbation in By component (Figure 8b) accompanied by changes in northward and then southward electric field (Figure 8c) are consistent with the observations as summarized in Figures 3d, 3h, and 3i, by taking into account the northward motion of the spacecraft relative to the current sheets (as presented in the arrow in Figures 8b and 8c).

Similar figures of the fields and flow patterns, but from a Hall‐MHD simulation by Nakamura et al. (2012), are shown in Figures 8d–8i from the Run 2 in their paper. In this run the reconnection starts from a current sheet with a half‐thickness of 1 di (ion inertial length) and from an 8 di wide region, located at *x* = 80 di and *y* = 75 di. The reconnection region then expands dawnward (−*y*) along the same direction as the current‐carrying electron motion. The snapshots shown in Figure 8 are after 55 ion gyro time, at which point a localized reconnection jet has developed well without being affected by the simulation boundaries. The jet properties shown here are downstream of the reconnection region by about 30 di, that is, ~3 R_E_ (for density value of 0.1/cc corresponding to beginning of this event). The simulation starts with a 1‐D current sheet configuration (without initial By and Bz components), which is in contrast to the large‐scale modeling of tail field and superposed dipole field setting by Birn et al. (2011). Nonetheless, the perturbation around the head of the reconnection jets still demonstrates the effects of the Hall field on the interactions between the localized reconnection jets and the ambient plasma as will be discussed below.

Figures 8d–8f show the *B*_*Y*_ perturbation in the *x*‐*z* plane at the dusk part of the ion jet and the *B*_*Y*_, and Ez disturbances in the *y*‐*z* plane, similar to the MHD simulation. The possible MMS crossing direction, which is in the southern hemisphere dusk portion of the flow in the *y*‐*z* plane, is indicated by a black arrow in Figures 8e and 8f and 8h and 8i. Due to the duskward tilt of the localized ion jet from the dawnward‐expanding reconnection region, a significant dawn‐dusk asymmetry is visible. Still we can identify a similar convection effect by the localized reconnection jet as in the MHD case, in addition to the Hall effects, as will be described below. *B*_*Y*_ perturbations in the *x*‐*z* planes (Figure 8d), taken from the dusk part of the flows, show an extension of the quadrupole field visible from the ion diffusion region at the poleward side (blue/red region in southern/northern hemisphere) and inverse Hall field due to jet braking in the equatorward side (red/blue region in southern/northern hemisphere). Here we call this pattern a 2‐D Hall effect of the reconnection jet. The *B*_*Y*_ perturbation created by the Hall effect, however, has the same perturbation signatures as that created from the localized flow in the +*y* region (duskside) as can be seen in Figures 8b and 8e along the black arrow. *B*_*Y*_ perturbation in the Hall‐MHD simulation (Figure 8e) shows that the 2‐D Hall effect is embedded in the localized flow effect, which results in also an asymmetric Ez pattern in the dawn‐dusk direction (Figure 8f). To separate these two effects: that is, contribution to Ez by the Hall and localized flow effects, Figures 8h and 8i present Eh_Z_ = −(Ve‐Vi) × *B* (Hall field) and −Vi × *B* (convection electric field), respectively. It can be seen that Figure 8i more closely resembles the MHD case (Figure 8c), while Figure 8h shows the quasi‐2‐D Hall electric field pattern as expected. When crossing the dusk and southern part of the boundary as indicated by the black arrows in the *y*‐*z* planes in Figure 8, the convection part of Ez (Figure 8i) has positive to negative variations, which is similar to the MHD case (Figure 8c). The Hall electric field part (Figure 8h) features only a strong positive value with the peak located near but equatorward of the positive peak of the convection Ez. Hence, the Hall electric field part looks to be embedded in the localized flow part in Figure 8f.

**Figure 9:**
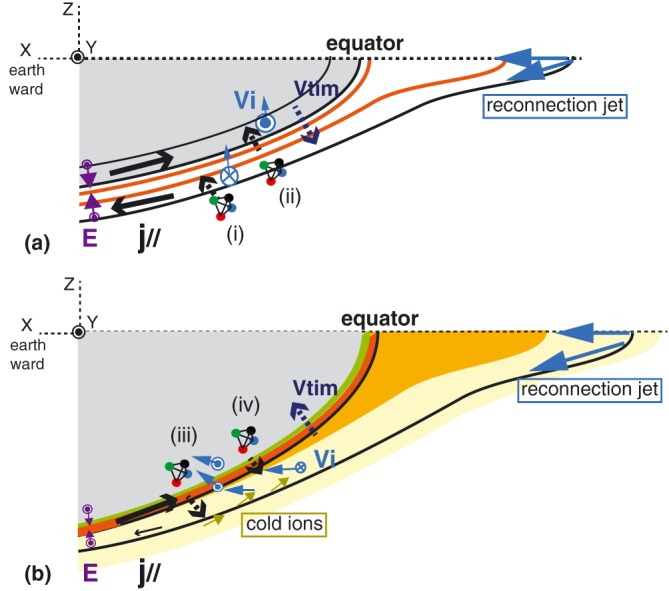
Schematics showing the main observational characteristics of (a) the field‐aligned current (FAC) events (i) and (ii) and (b) the FAC events (iii) and (iv) and the ion‐scale thin (Hall) current layers flowing duskward (red) and dawnward (light green). FAC directions are given by the black arrows. The dotted arrow shows the spacecraft motion relative to the current sheet; that is, the motion of the current sheet itself is outward for the events (i) and (ii) and inward for events (iii) and (iv). The ion bulk flow is given in blue arrows, while the purple represents the electric field direction. The light yellow color in (b) represents region with tailward cold ion beams.

The observed characteristics of the FAC events (i) and (ii) and the embedded Hall current layers are summarized in the drawings in Figure 9a. While the observations do not conform to such a simple pattern as the simulation, the stronger equatorward electric field (+*E*_*H*_) compared to the outward electric field (−*E*_*H*_) shown in Figure 2j is consistent with the prediction from the Hall‐MHD simulation on the duskside of the reconnection jet, even though the structures feature larger dawn‐dusk asymmetries than the MHD simulation. Also, the small‐scale embedded Hall current sheets observed between the events (i) and event (ii), as discussed in Figures 3, 5, and 6, indicate that the region of the equatorward electric field (where Hall effect to be expected) consists of ion‐scale boundaries similar to that in the Hall‐MHD simulation. The Hall effect is dominant in the localized reconnection simulation by Nakamura et al. (2012). The observations also obtained larger Hall electric field than the convection electric field, whereas the relative importance was quite variable depending on the types of the current sheet as discussed in Figure 5.

The scale size of the current layers of events (iii) and (iv) is, on the other hand, comparable to or below the ion inertial length and larger than the ion‐gyro scale. As expected for this spatial scale size, the VDF (Figure 7) showed features of unmagnetized ions, moving along the electric field direction. Figure 9b illustrates the observational characteristics of the FAC events (iii) and (iv). Further differences from the events (i) and (ii) are that the current sheet motion was inward, that is, in the same direction as the convection. The crossing of the FAC layers (iii) and (iv) are followed by the appearance of the hot Earthward‐moving ion beams, together with the cold ions and electrons as also shown in Figure 7. These cold ions are streaming tailward and drifting with *E* × *B* drift. Such cold ions have been identified in PSBL; they are streaming from the ionosphere and become visible when the electric field is sufficient to gain *E* × *B* energy above the detector energy range (Sauvaud et al., 2004). One should, however, note that the cold ions are only visible following the FAC events (iii) and (iv), but not during events (i) and (ii), when the *E* × *B* drift should have been even larger. Hence, for the case we studied, the cold ions seemed to exist only on the flux tubes MMS encountered during events (iii) and (iv). We suggest that the events (iii) and (iv) are the inner boundary of the equatorward/inward convecting flux tubes resulting from reconnection involving lobe or PSBL field lines with enhanced ionosphere outflows related to global wind (Moore & Horwitz, 2007) or due to direct ion injection (Sauvaud et al., 2004). In the direct injection case, however, the time scale of such ion upward accelerations is larger than 10 min to be observable at MMS. That is, the sudden encounter of the cold ions is not due to the sudden injection itself but rather due to encounter of the flux tubes already filled with the cold ions as illustrated in Figure 9b. This scenario, where multicomponent ions generate a Hall current in the exhaust far downstream of the reconnection *x*‐line, has been studied in numerical simulations (Fujimoto & Takamoto, 2016; Higashimori & Hoshino, 2012). In such Hall current exhaust, the ion drift velocity is modified around the current layer due to a finite Larmor radius effect (Fujimoto & Takamoto, 2016). Our observation also showed such Hall current layer with the mixture of unmagnetized hot ions and magnetized cold ions *E* × *B* drifting together with electrons as given in Figure 7g (*t*_*G*_) during event (iv) as sketched as red region in Figure 9b. It is interesting to note that the most unmagnetized distribution of ions was found at the beginning of event (iv) as shown in Figure 7g (t_F_) and illustrated in Figure 9b as a green flux tube. It corresponds to the strongest dusk‐to‐dawn currents (Figure 5). During this time, the ions tend to move along −Vperp2, that is, Earthward/equatorward, as if keeping the original motion of the reconnection jet rather than drifting with *E* × *B*. These features are consistent with the mechanism responsible for creating the opposite Hall field at the head of the reconnection jets where unmagnetized ions overtake the magnetized electrons, which has been shown in numerical simulations (e.g., Nakamura et al., 1998). The resultant magnetic field disturbance, opposite to those in the ion diffusion region near the *x*‐line, can be also seen at the equatorward side of the leading part of the jet in Figure 8d (as discussed before). The best agreement with electron perpendicular motion and *E* × *B* drift was achieved for the thin perpendicular currents during event (iv) among all the thin currents (Figure 7d). During event (iv), therefore, only the magnetized electron drift contributes to the Hall current.

While we have so far concentrated on processes with ion scales or larger, the above description may need some modification once shorter time variations are taken into account. For example, the first type of Hall current described above contains highly structured electron currents due to interactions with lower‐hybrid waves, which was obtained based on the high‐resolutions particle and field data (Le Contel et al., 2017). In the presence of high‐frequency wave activity, our averaged data processed beyond ion‐gyro time scales may smear out the responsible electric fields and may cause differences between the *E* × *B* drift and the electron motion in addition to the possible contribution from electron‐scale processes. Such wave activity was, however, weaker for event (ii) (Le Contel et al., 2017). The overall agreement between the electric‐field drift and electron perpendicular velocity shown in Figure 3, and the agreement between the curlometer current and the particle current during the intense dawn‐dusk current events indicated in Figure 5, suggested that the overall processes of these intense current events can be well described with Hall physics once different ion components are taken into account.

The four FACs have also differences in the nonthermal signatures of electrons. All the fronts are accompanied by the enhancements in the electron energy: Events (i) and (ii) are associated with enhancements in parallel/antiparallel component, while events (iii) and (iv) are associated with appearance of an enhanced perpendicular population. Previous observations of dipolarization fronts in the inner plasma sheet also showed energetic electron enhancements of different pitch angle population (Fu et al., 2011; Runov et al., 2013). The two types of populations have been interpreted to be due to different electron acceleration mechanisms: Fermi and betatron mechanisms. Fu et al. (2011) suggested that the pancake‐type (maximum in 90° pitch angle) PADs appear mainly inside the growing FPR where the flow velocity is increasing and the local flux tube is compressed. The cigar‐type (maxima at 0° and 180° pitch angle) PADs occur mainly inside the decaying FPR, where the flow velocity is decreasing and the local flux tube is expanding (flow braking). Runov et al. (2013) reported that pancake‐type and cigar‐type PADs coexist at the dipolarization front and that the PADs are mainly pancake type near the neutral sheet (Bx < 5 nT) and mainly cigar type outside (Bx > 10 nT). Both observations are from dipolarization fronts relatively near to the center of the plasma sheet, which is different from our observations. Yet there are certain similarities to our observations in the outer plasma sheet. As discussed before, events (i) and (ii), which contain cigar‐type PADs, are likely associated with flow braking as suggested by Fu et al. (2011) and away from the neutral sheet (Runov et al., 2013). While events (iii) and (iv), which observed pancake‐type PADs, took place not in the neutral sheet, but the field is in a more dipolar configuration (larger Bz) and it is at the inner edge of exhaust, which are similar condition to the pancake PAD observations by Runov et al. (2013) and by Fu et al. (2011). Hence, we may have detected equivalent acceleration processes to those observed in the dipolarization front at the center of the plasma sheet. It is interesting to note that these different types of the fronts were sequentially detected within a relatively short time in our observation.

## 6 Conclusions

Magnetospheric Multiscale resolved the characteristics of intense current layers within the dusk part of the SCW of a substorm during fast flow disturbances (up to about 500 km/s), mostly in the dawn‐dusk direction. MMS first encountered downward and upward field aligned currents associated with expansion of the plasma sheet followed by upward field aligned current layers on the thin equatorward convecting flux tubes, preceding PSBL‐type Earthward streaming hot ions and tailward streaming cold ions. The FACs are initially associated with expansion of the plasma sheet, and the flow and field disturbances showed distinct pattern expected in the braking region of localized flows. Supporting evidence was found in the simulated signatures of reconnection jets.

Intense Hall current layers were found adjacent to the FACs. Three types of ion distributions in the Hall currents were found: (1) nearly magnetized ions moving slightly slower than the *E* × *B* drift, (2) a mixture of magnetized cold ions and unmagnetized hot ions, and (3) unmagnetized hot‐ion distributions. The most intense Hall current, flowing dusk to dawn, was associated with the type (3) that took place at the inner edge of an upward FAC layer at the front of the hot Earthward streaming ions, possibly associated with the front of the reconnection jet. The largest Hall electric field observed during the entire interval was about twice as large as the maximum convection electric field.

These observations showed that field aligned currents in the flow braking region are multiscale processes involving processes relevant to reconnection jet from thin current sheet and the evolution of the localized flow vortices. The mixing of the hot ions streaming Earthward from the reconnection jet and the tailward moving cold ion components affects the Hall current processes near the dipolarization front. It is important to take into account these multiscale multicomponent plasma processes to understand the evolution of the SCW.
